# Cost-analysis of universal decolonization with pure hypochlorous acid and mupirocin to reduce MRSA infections in intensive care burn unit patients

**DOI:** 10.3389/fphar.2025.1606589

**Published:** 2025-06-17

**Authors:** Peter J. Mallow, Debashish Chakravarthy, Kevin Foster

**Affiliations:** ^1^ Department of Health Services Administration, Xavier University, Cincinnati, OH, United States; ^2^ Urgo Medical North America, Dallas, TX, United States; ^3^ Arizona Burn Center, Valleywise Health, Phoenix, AZ, United States

**Keywords:** cost-analysis, MRSA, burns, PHA, infections

## Abstract

**Introduction:**

Burn patients are at high risk for infections, particularly Methicillin-resistant *Staphylococcus* aureus (MRSA). Universal decolonization strategies have demonstrated effectiveness in reducing infection rates. This study aimed to evaluate the cost implications of using pure hypochlorous acid (pHA) and mupirocin to prevent MRSA infections in hospitalized burn patients.

**Methods:**

A patient-level microsimulation model was developed to perform a cost analysis from the US health system perspective. Clinical inputs were derived from a retrospective observational study. The primary outcome was the reduction in MRSA infections per 1,000 bed days. Cost estimates, expressed in 2023 US dollars, were gathered through a pragmatic literature review of publicly available sources. Deterministic and probabilistic sensitivity analyses were conducted to assess the robustness of the findings.

**Results:**

Before the introduction of pHA, burn patients were 3.05 times more likely to develop MRSA infections. The estimated cost of treating MRSA infections was $224,376 per 1,000 bed days in the pre-pHA period, compared to $148,812 in the post-pHA period. After including the cost of pHA, the net savings amounted to $75,564 per 1,000 bed days, or $75.56 per bed day. Sensitivity analyses confirmed the robustness of these results across a range of input values.

**Conclusion:**

The combination of pHA and mupirocin appears to be a cost-saving strategy for reducing MRSA infections among hospitalized burn patients.

## Introduction

The care for burn patients has improved greatly over the past 10 years; however, infections remain a significant and burdensome complication. Infections account for 42%–65% of all deaths in burn patients (Bloemsma et al., 2008; Gomez et al., 2009). Methicillin-resistant *Staphylococcus aureus* (MRSA) is of particular concern regarding burn patients (Samuel et al., 2023). MRSA is positively associated with increased length of stay in burn patients and up to 70% of patients may be colonized with MRSA after 3 weeks in the hospital (Randa and Abdelrahman, 2024). Universal decolonization has been shown to reduce MRSA infection rates (Hacek et al., 2009). Efforts to decolonize include screening, isolation of patients, daily cleaning and disinfecting of surfaces, as well as the universal use of mupirocin and chlorhexidine (Randa and Abdelrahman, 2024; Gray et al., 2016). However, the use chlorhexidine in burn patients is subject to increased risk of chemical burns (Abdel-Sayed et al., 2020). The use of pure hypochlorous acid (pHA) has been found to be effective in reducing MRSA on the skin and controlling bacterial bioburden (Robson et al., 2007; Mallow et al., 2024). However, the economic implications of using pHA to prevent MRSA infections is not well understood. The objective of this study was to conduct a cost-analysis of pHA and mupirocin for the prevention of MRSA infection in hospitalized burn patients.

## Data and methods

### Data

All clinical data were obtained from a retrospective observational study from a single center, Arizona Burn Center at Maricopa Medical Center (Gray et al., 2016). The clinical data examined the admitted burn patients for a 1-year period prior (2013) and 1 year period post (2014) introduction of pHA and mupirocin. All data were retrieved retrospectively from the electronic medical records. Decolonization practices consisted of enhanced cleaning and disinfection practices, hand hygiene, universal contact precautions, and ultraviolet light disinfection. During the pre-intervention period, the standard of care included daily bathing with a 2%–4% chlorhexidine solution and a 5-day course of 2% nasal mupirocin. The post-intervention period consisted of pHA moistened dressings, volume of pHA was determined by clinician, instead of the chlorhexidine solution. The primary outcome variable was the reduction in MRSA infections per 1,000 bed days. The intervention decreased infection rates from 7.23 per 1,000 patient days to 2.37 per 1,000 patient days (3.05 times less likely to acquire a MRSA infection) (Gray et al., 2016). Cost data were obtained from the publicly available data sources in 2023 United States Dollars using a pragmatic literature review. The pragmatic literature review consisted of a literature search of English language articles from 1 January 2020 to 31 December 2023 for costs associated with MRSA infection and pHA and mupirocin utilization.

### Methods

A patient-level microsimulation model was used to conduct a cost-analysis from the US health system perspective. The model assessed the expected costs associated with MRSA with and without the use of pHA and mupirocin. The difference between the two strategies was used to ascertain the net effect of introducing pHA to prevent infections in burn patients per 1,000 days. The model development was informed by the International Society of Pharmacoeconomics and Outcomes Research (ISPOR) good research practices (Roberts et al., 2012). Deterministic and probabilistic sensitivity analyses (PSA) were performed to gauge the robustness and reliability of the results. The deterministic sensitivity analysis varied each parameter by the low and high values to assess the influence of each individual parameter. The low and high value varied ± 25 percent for cost variables and ± 10 percent for the infection rates. The PSA used a Monte-Carlo approach to calculate the expected costs for each strategy by varying the parameters by the distributions listed in Table 1 for 10,000 simulated patients. The model was developed using Treeage Software (Williamstown, MA). The base case results were verified through the development of identical model in Excel (Microsoft, Redmond, WA). The model relied on de-identified publicly available data was exempt from Institutional Review Board review.

**TABLE 1:** Model parameters.

Parameter	Values			Distribution	References
	Base	Low	High		
Pre-Infection Rate/1,000 days	7.23	5.42	9.04	Normal	Gray et al. (2016)
Post-Infection Rate/1,000 days	2.37	1.78	2.96	Normal	Gray et al. (2016)
Cost of MRSA Infection	$30,988	$23,241	$38,735	Triangle	Nelson et al. (2021)
Cost of pHA/day	$75	$56	$94	Triangle	Mallow et al. (2024)
Cost of mupirocin/day	$6.8	$5.1	$8.5	Triangle	(Woodard)

This table presents the parameter values used in the patient-level microsimulation model. The base case reflects the typical estimate, while the low and high values represent the range of uncertainty. The distribution indicates the statistical form applied in the simulation, and the reference cites the data source.

## Results

**FIGURE 1:**
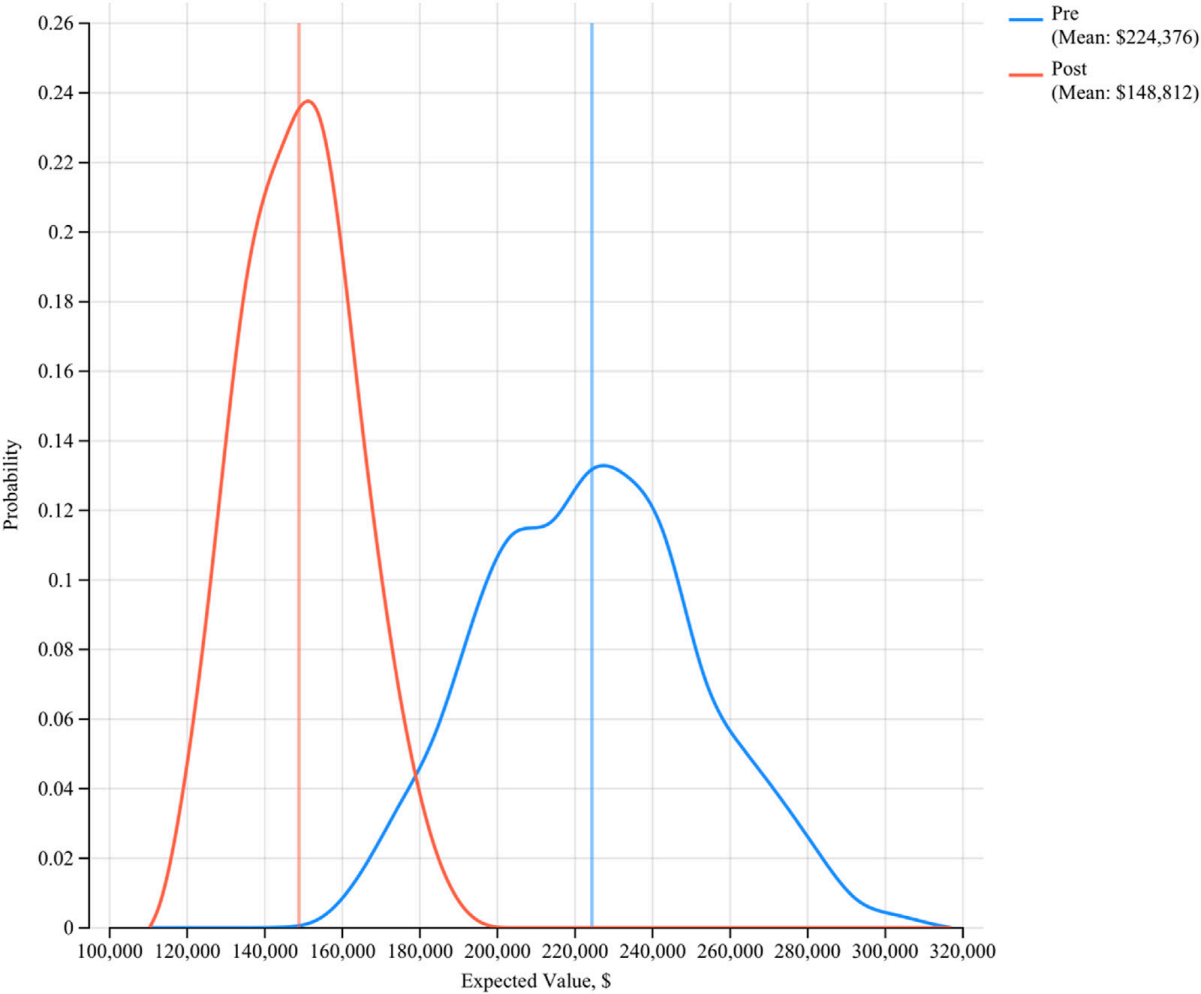
Expected Costs Pre-/Post-pHA and Mupirocin. The figure shows the expected value distribution from the patient level microsimulation model. The red lines represent the post-intervention results. The blue lines represent the pre-intervention results.

**FIGURE 2:**
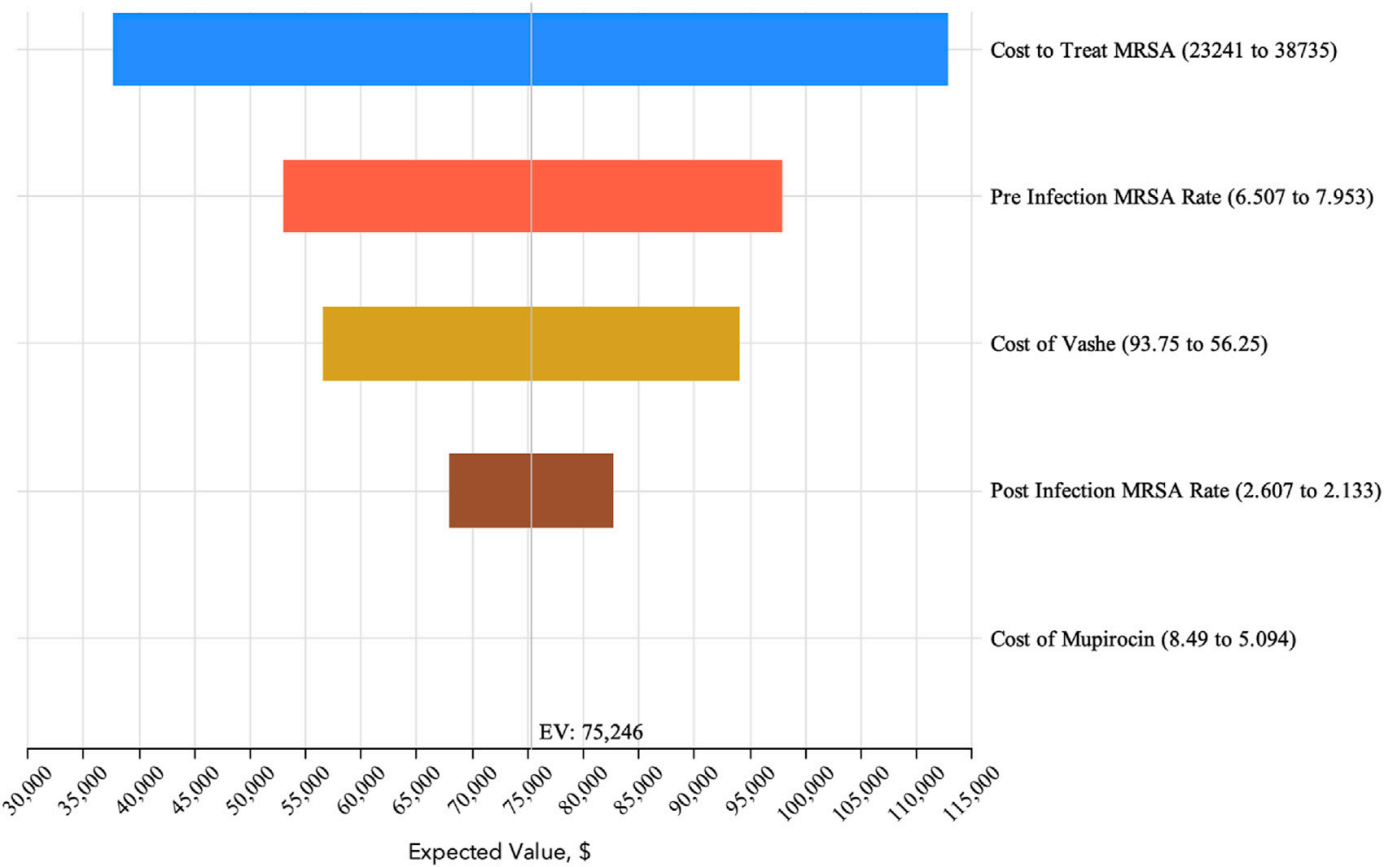
One-Way Sensitivity Analysis. The one-way sensitivity analysis visually shows variation in the expected value based upon varying individual parameters based upon the ranges reported in Table 1.

The expected cost to treat MRSA infections in the pre-pHA period was $224,376 per 1,000 bed days, whereas the expected cost in the post-pHA period was $148,812 per 1,000 patient bed days (Figure 1). The net savings associated with the addition of pHA and mupirocin was $75,564. The PSA revealed that pHA and mupirocin were expected to save the hospital money 100 percent of the time. The expected savings ranged from $1,258 to $183,115 based on varying the model parameters. The one-way sensitivity analysis revealed that the cost to treat a MRSA infection had the most influence on the results. The expected savings ranged from $37,000 to $112,000 per 1,000 bed days (Figure 2).

## Discussion

The findings of this study demonstrate that the use of pHA and mupirocin for decolonization in burn patients led to substantial cost savings. MRSA infections substantially increase the length of stay, resource utilization, and overall treatment expenses. By reducing MRSA infections, hospitals can lower antibiotic use, improve bed turnover, increase patient satisfaction and reduce clinical time per patient. The clinical study relied upon for this economic analysis found a 3-fold decrease in MRSA infections between the pre-pHA and post-pHA period. (Gray et al., 2016). The expected savings of $75,564 over a period of 1,000 bed days after accounting for the added cost of pHA and mupirocin equate to $75.56 per day. In a capitated payment model for inpatient burn care, the expected savings is a direct financial benefit to the hospital.

### Limitations

All economic analyses are subject to limitations. First, the results of this analysis were based on a single center burn unit’s experience with pHA and mupirocin in a pre-/post-period observational study. As such the results may not be generalizable to other settings. Second, this study was limited only to an examination of intervention effect on MRSA. It did not include an assessment of the cost of all treatment, ancillary burn-related services, or labor productivity related to the intervention. Despite these limitations, these results provide insight into the economic effectiveness of decolonization with pHA and mupirocin in burn patients. The relatively minor cost of adding pHA and mupirocin to the treatment protocol is more than addressed by the anticipated savings of fewer MRSA infections.

## Conclusion

The addition of pHA and mupirocin to the treatment protocol for burn patients was shown to be a cost saving strategy in the reduction of MRSA infections. The adoption of pHA and mupirocin should be considered a value-added adjunct therapy in the treatment of burn patients.

## Data Availability

The raw data supporting the conclusions of this article will be made available by the authors, without undue reservation.
